# Identification and Characterization of Key Genes Responsible for Weedy and Cultivar Growth Types in Soybean

**DOI:** 10.3389/fgene.2022.805347

**Published:** 2022-02-24

**Authors:** Prakash Basnet, Taeyoung Um, Neha Samir Roy, Woo Suk Cho, Soo Chul Park, Kyong-Cheul Park, Ik-Young Choi

**Affiliations:** ^1^ Department of Agriculture and Life Industry, Kangwon National University, Chuncheon, South Korea; ^2^ Department of Agricultural Biotechnology/National Academy of Agricultural Science, Rural Development Administration, Jeonju, South Korea

**Keywords:** weedy type, cultivar type, transcriptome, differentially expressed genes, phytohormone

## Abstract

In cultivated plants, shoot morphology is an important factor that influences crop economic value. However, the effects of gene expression patterns on shoot morphology are not clearly understood. In this study, the molecular mechanism behind shoot morphology (including leaf, stem, and node) was analyzed using RNA sequencing to compare weedy (creeper) and cultivar (stand) growth types obtained in F_7_ derived from a cross of wild and cultivated soybeans. A total of 12,513 (in leaves), 14,255 (in stems), and 11,850 (in nodes) differentially expressed genes were identified among weedy and cultivar soybeans. Comparative transcriptome and expression analyses revealed 22 phytohormone-responsive genes. We found that *GIBBERELLIN 2-OXIDASE 8* (*GA2ox*), *SPINDLY* (*SPY*), *FERONIA* (*FER*), *AUXIN RESPONSE FACTOR 8* (*ARF8*), *CYTOKININ DEHYDROGENASE-1* (*CKX1*), and *ARABIDOPSIS HISTIDINE KINASE-3* (*AHK3*), which are crucial phytohormone response genes, were mainly regulated in the shoot of weedy and cultivar types. These results indicate that interactions between phytohormone signaling genes regulate shoot morphology in weedy and cultivar growth type plants. Our study provides insights that are useful for breeding and improving crops to generate high-yield soybean varieties.

## Introduction

Soybean [*Glycine max* (L.) Merr.] is a major species of legume crop grown for human and animal consumption. Although *Glycine soja* (wild soybean) is genetically close to cultivated *G. max*, it is a divergent phenotype (Singh and Hymowitz, 1988; Stupar, 2010; Joshi et al., 2013; Kofsky et al., 2018). The cultivated soybean has an erect growth type, a strong primary stem, and large variable seeds, whereas the wild soybean is a creeper and has a weedy growth type, a long and thin vine stem, and small seeds (Liu et al., 2007). Soybean plants morphology varies according to its domestication characteristics which are wild, semi wild, and cultivated soybeans (Qiu et al., 2014). Agronomical traits are influenced by stem growth habit. According to stem termination, soybean growth types are classified into determinate as the cultivar type and indeterminate, the weedy type (Bernard, 1972). In weedy types, the vegetative activity continues with the terminal bud resembling long and thin stem (Liu et al., 2010). In major crops, shoot morphology is the prime factor for grain yield (Kurepin et al., 2013; Chen et al., 2020; Zhang et al., 2021); desirable vertical shoot structure with beneficial traits, plant height, leaf size and stem structure lead to improved economic return (Khush, 2001; Mencuccini, 2015). (Kilgore-Norquest and Sneller, 2000) reported that cultivar soybeans had greater yield than the wild soybeans at the late planting dates. Therefore, unraveling the genetic mechanism behind plant architecture aids the development of high-yield cultivars (Wang and Li, 2008).

Development and defense systems in plants are mainly controlled by plant hormones (Bari and Jones, 2009; Huot et al., 2014; Davière and Achard, 2016). Phytohormones also regulate plant morphology (Hepworth and Lenhard, 2014; Yue et al., 2016). The major plant hormones viz, gibberellin, auxin, and cytokinin influence cell differentiation, proliferation, and elongation in plants and are responsible for divergent shoot architectures (Mok, 1988; Gaspar et al., 1996; Azizi et al., 2015; Haruta and Sussman, 2017). Gibberellin regulates stem elongation in plants (Jones and Kaufman, 1983; Harberd et al., 1998; Kende et al., 1998; Olszewski et al., 2002). Gibberellic acid (GA) also commonly called gibberellins, a tetracyclic di-terpenoid compound stimulating plant hormone (Gupta and Chakrabarty, 2013). Deregulation of GA-responsive genes, gibberellin-oxidases (*GA20oxs*, *GA3oxs*, and *GA2oxs*)results in altered phenotypes in plants (Thomas and Sun, 2004; Dai et al., 2007; Rieu et al., 2008; Wuddineh et al., 2015; Wang et al., 2017). Moreover, *GA20ox* and *GA3ox* promotes bioactive GA through the biosynthetic signaling pathway (Busov et al., 2008; Yang et al., 2012) whereas *GA2ox* involves in inactivation of GA (Thomas et al., 1999; Olszewski et al., 2002). Functional deficit of *GA20x* and *GA3ox* results in semi dwarf plants in *Arabidopsis* (Yamaguchi et al., 1998) and rice (Sakamoto et al., 2001). However, mutants of *GA2ox* responses opposite of *GA20ox* and *GA3ox*, resulting long and slender stem in pea (Martin et al., 1999) and switchgrass (Wuddineh et al., 2015). In soybean, *Glyma18g06870* (*GAox*), 2-oxoglutarate-dependent dioxygenase is identified as a candidate gene responsible for vine growth habit (VGH) (Wang et al., 2019).

Shoot organogenesis and overall plant architecture is also determined by auxin (Gallavotti, 2013; Taylor-Teeples et al., 2016). Auxin response factors (*ARFs*) and Indole Acetic acid (*IAA*) plays an important role in regulation of auxin response genes (Ma and Li, 2019). ARF transcription factor interacts with PHYTOCHROME INTERACTING FACTOR (PIF) to increase auxin biosynthesis and promote cell elongation in the stem of *Arabidopsis* (Oh et al., 2014)*.* All physiological mechanisms in *A. thaliana*, such as cell division, shoot apical dominance, tropism, and root initiation, are governed by auxin (Hagen and Guilfoyle, 2002). Upstream of auxin responsive genes promote cell elongation of the hypocotyls and plant organs (Chae et al., 2012). Shoot apical balance is controlled by cytokinin via the regulation of cell division and cell proliferation (Mok and Mok, 2001). The level of active cytokinin is suppressed by cytokinin dehydrogenase (CKX) (Motyka et al., 1996; Werner et al., 2001). For example, high regulation of *CKX* gene in *Arabidopsis* inhibit cytokinin level, indicating a decrease in overall shoot morphology (Schmülling et al., 2003). Three cytokinin receptors (*ARABIDOPSIS HISTIDINE KINASEs; AHK2, 3, 4*) and eleven response regulators (*ARABIDOSPIS RESPONSE REGULATORs*, *ARRs*) have been identified in *Arabidopsis* determining the specificity of cytokinin in plant growth (Kim et al., 2006). The receptor gene *AHK* involve in activating cell division and meristem maintenance (Jeon et al., 2010).

Interaction among plant hormones controls overall plant growth and development (Ohri et al., 2015). Auxin promotes bioactive gibberellins like gibberellic acid-1 (GA1), and is most important for cell division and internode elongation (Ross et al., 2001). Both cytokinin and gibberellin act mutually in the early and late developmental stages of shoot meristem elongation (Richards et al., 2001; Schmülling, 2002). Auxin interacts with cytokinin antagonistically. For example, apical dominance is regulated by auxin and cytokine in an antagonistic manner (Shimizu-Sato et al., 2009). Reduction of auxin signaling gene expression induces cytokinin signaling in most tissues (Kurepa et al., 2019).

Growth and development are continuous processes influenced by phytohormone interactions that produce diverse phenotypes. To date, few studies have been conducted to explore the genes that determine the weedy and cultivar growth types in soybean. Comparative transcriptome analysis by RNA sequencing is an efficient method for identifying important differences in gene expression between two plant relatives (Koenig et al., 2013). Here, we used the transcriptomes of leaves, stems, and nodes to identify differentially expressed genes (DEGs) associated with the weedy and cultivar growth types in soybean. We performed high-throughput Illumina sequencing to comprehensively characterize the transcriptomes. To investigate how the expression of these genes affects soybean phenotypes, we compared the expression profiles of the leaves, stems, and nodes and the crosstalk of DEGs. The results of this research provide insights into the relevant genes responsible for the weedy and cultivar growth types in soybean and facilitate a better understanding of the molecular mechanism behind the differences in growth and development of the two soybean relatives.

## Materials and Methods

### Plant Materials

KB000001 (*Glycine max*), a cultivated soybean and KB000002 (a hybrid of *G. max* and *Glycine soja*), a wild type of soybean seeds was collected from Rural Development Administration (RDA), South Korea. The parents, KB000001 with an erect phenotype and KB000002 with the weedy phenotype, were crossed to generate recombinant inbred lines (RILs) by single seed descent (SSD) method. A single was obtained from the first filial generation (F_1_) grown in the field in Chuncheon-Si (Gangwon-do, South Korea). To generate an inbred population, the F_2_ seeds were harvested and grown by selfing further until the F_5_ generation. Both the weedy and cultivar growth types were observed in every filial generation. From F_1_ to F_6_, two lines were targeted, with each displaying one of the distinct phenotypes of the parental lines, i.e., the weedy (creeper) and the cultivar (stand) growth types. The selected plant’s seeds from F_6_ were harvested and grown both in the field and the greenhouse. The shoot morphology in F_7_ was the same as F_6_, so they were selected for the rest of the study. To confirm the phenotype, the plants were grown in the growth room with optimal temperature (25°C) and 60–70% humidity. Finally, these two lines were selected to study the molecular mechanism underlying the weedy and cultivar growth types. The leaves, stems, and nodes from 12-week-old plants were sampled and harvested immediately in frozen liquid nitrogen (N) for RNA isolation.

### RNA Extraction and RNA-Seq Library Construction

Total RNA was isolated from the leaves, stems, and nodes from the respective samples using GeneAll® Ribospin™Plant (Geneall Biotechnology Co., Ltd., Seoul, Korea) following the manufacturer’s instructions. Genomic DNA digestion was performed with DNase I (Sigma, St. Louis, MO, United States of America). The RNA quality was observed on 1% agarose gel electrophoresis. The total RNA integrity was checked using an Agilent 2100 BioAnalyzer with RNA integrity number (RIN) value >6. For the total mRNA library, sequencing was performed using the Illumina Nova Seq high-throughput sequencing kit and platform (Illumina Ltd, San Diego, CA, United States) according to the manufacturer’s protocol and performed by Theragen Etex Bio Institute, a professional DNA sequencing service provider (Theragen Etex Inc. Suwon, South Korea).

### Screening of Differentially Expressed Genes by RNA-Seq

The sequenced data from Illumina were preprocessed using Trimmomatic in which raw data were trimmed and converted to total read bases. The adapters were used for trimming contaminant sequences and for library construction in Trimmomatic v0.36 (http://www.usadellab.org/cms/index.php?page=trimmomatic). Low-quality reads were removed by applying Trimmomatic’s sliding window (4:20) with average quality (30) and minimum read size (36 bp). The sequence was read in units of 4 bp with a PHRED score less than 20. Reads with average length of less than 20 bases were removed. Each sample was aligned to the reference sequence, *Glycine max* Wm82.a2. v1, using the HISAT2 v2.1.0 program (https://ccb.jhu.edu/software/hisat2/index.shtml). The processed sequences consisting of paired-end reads were mapped to the reference sequence. The read count (expression level) was calculated using StringTie v1.3.4d software (https://ccb.jhu.edu/software/stringtie/index.shtml). Based on the read count of the StringTie calculated at the transcript level, a comparative analysis between the samples was performed using DESeq V1.36.0. In DESeq, read count was normalized through size factor and dispersion, DEG analysis was performed using a log_2_ fold change value, and false discovery rate (FDR) was calculated after normalization. Calculation of unigene expression was performed using the fragments per kilobase of transcript per million mapped reads (FPKM) method. FPKM values were calculated for each of the genes and only unigenes with FPKM >1.0 were used for the further analysis. Visualization analyses, i.e., heat maps, Venn diagrams, and Minus versus Add (MA) plots, of the DEGs were performed by an in-house R script.

### Gene Annotation and Functional Analysis

The genomic reference information of *Glycine max* was obtained from Soybase, which includes Gene Ontology (GO) annotations for each gene. The Kyoto Encyclopedia of Genes and Genomes (KEGG) annotations were obtained from the KEGG database. The BLASTp (e-value 1e-3) analysis was performed using NCBI’s Refseq (http://www.ncbi.nlm.nih.gov) plant protein sequence, TAIR, and Uniprot as databases (DB). Gene Ontology analysis with biological process, cellular component, and molecular function was done using InterProscan and BLAST. The KEGG pathway was performed by BLAST2GO (https://www.blast2go.com/). A *p*-value of ≤.05 was used as a significance threshold for the GO and KEGG pathway enrichment analysis.

### Reverse-Transcription Quantitative PCR Analysis

RT-qPCR analysis was conducted using the SyBr Green PCR kit on a PIKOREAL 96 Real-Time PCR system to validate RNA-Seq data. Candidate reference RT-qPCR gene primers were designed using IDT (Integrated DNA technologies). The *GmAct6* (Actin-6) soybean gene was selected as the endogenous reference. All the primers were synthesized by Bionicsro.co.kr. One-step RT-qPCR analysis was performed on the extracted total RNA in three biological replicates and three technical replicates of the leaves, stems, and nodes. A total of 10 μl RNA was used for cDNA synthesis using the HyperScript™ Rt master mix (with oligo dT) protocol for RT-qPCR expression analysis. In this experiment, each reaction contained 2 µl of diluted cDNA, 1 µl of each primer, 5 µl of 2X SYBR Green mix, and 2 µl of RNase free water. All the RT-qPCRs were performed with the following conditions: denaturation at 95°C for 1 min, followed by 40 cycles of 95°C for 5 s, 57°C for 10 s, 72°C for 10 s, and final extension at 72°C for 5 min. To verify product specificity, melting curve analysis was performed after each amplification. The relative expression level of each unigene was calculated using the 2^−ΔΔCT^ approach (Livak and Schmittgen, 2001).

## Results

### Phenotype Selection During Generation of the Recombinant Inbred Lines

**FIGURE 1:**
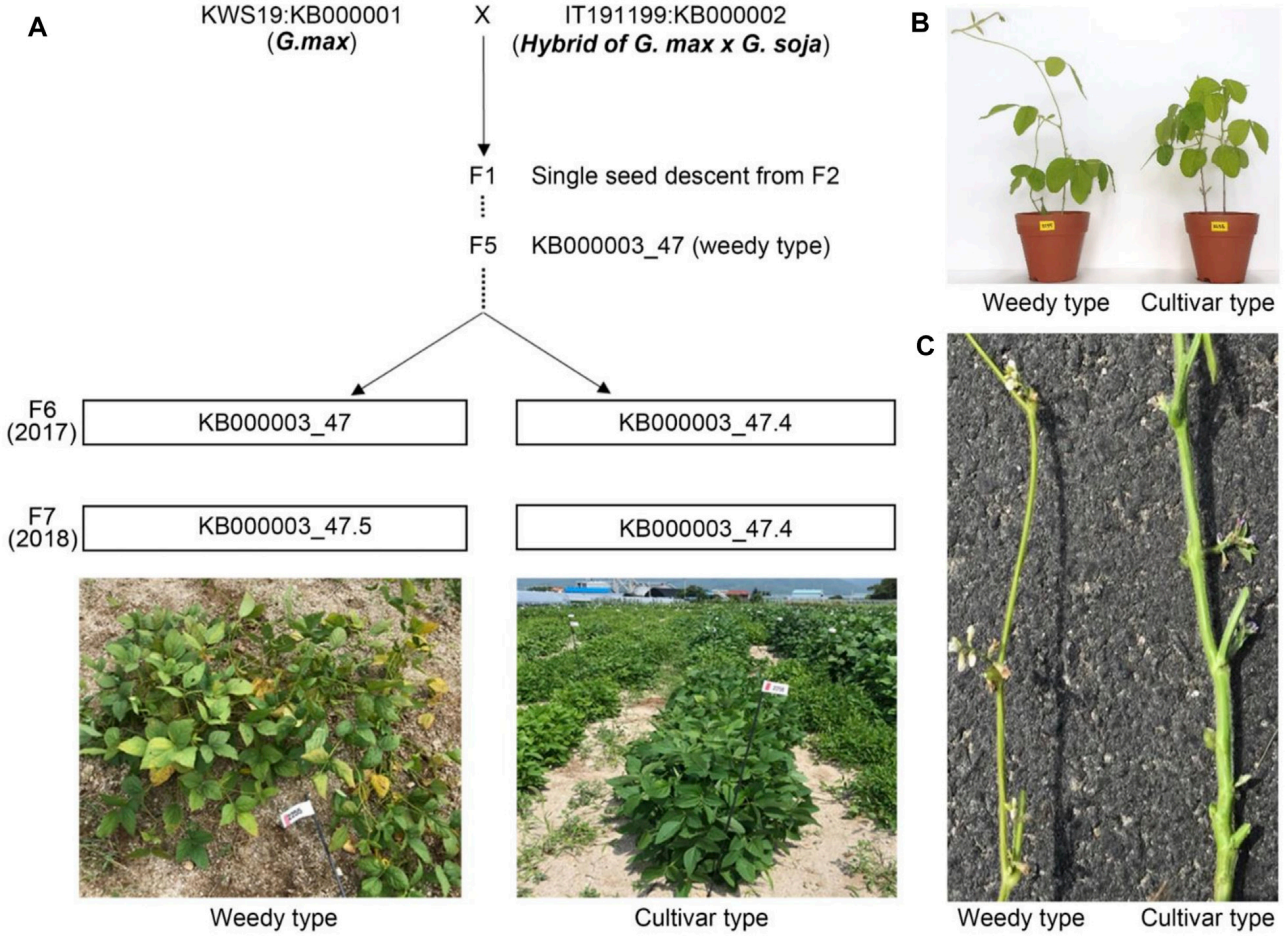
Population phenotypes and crossing scheme used to generate the recombinant inbred lines (RILs). **(A)** Standard parents *G. max* (KWS: KB000001) and a hybrid of *G. max* and *G soja* (KWS: KB000001) were used for crossing, and their phenotype distribution descended from F2 to F7. Individual lines from F6 displaying parental phenotype, i.e., weedy (KB000003_47.5) and cultivar type (KB000003_47.4), continues in F7. **(B)** Morphology of weedy and cultivar growth type plants (F7 generation). Eight-week-old plants were grown in a greenhouse. **(C)** The stems of weedy type plants and cultivar type plants have different morphology. The F7 generation weedy and cultivar type plants were grown in the field under normal conditions.

To understand phenotypic variation among two plant relatives at the transcriptome level, we crossed representative parent soybeans, cultivated type and a wild type, to generate recombinant inbred lines (RILs) (Figure 1A). We obtained independent RILs over seven generations by selfing. The F_2_–F_5_ generation plants, generated using single seed descent from F_1_, could be segregated by plant height, maturing time, and plant structure (weedy and cultivar types). We generated F_6_ plants with the weedy phenotype from KB000003-47 RIL (F_5_). We obtained F_7_ seeds from a weedy type of plant (KB000003_47) and a cultivar type plant (KB000003_47.4) in F_6_. In the F_7_ generation, the phenotype of plants did not differ between normal field and greenhouse conditions (Figures 1A,B). The weedy type (KB000003_47.5) typically showed a thin stem, leaf senescence, and reduced node number compared to the cultivar type (KB000003_47.4). In all the generations (F_6_-F_7_), there were clear differences in stem morphology between the weedy type and cultivar type (Figure 1C). These results indicate that the stem can be used to characterize developmental differences among weedy type and cultivar type plants.

### Assembly of RNA Sequencing Data

**FIGURE 2:**
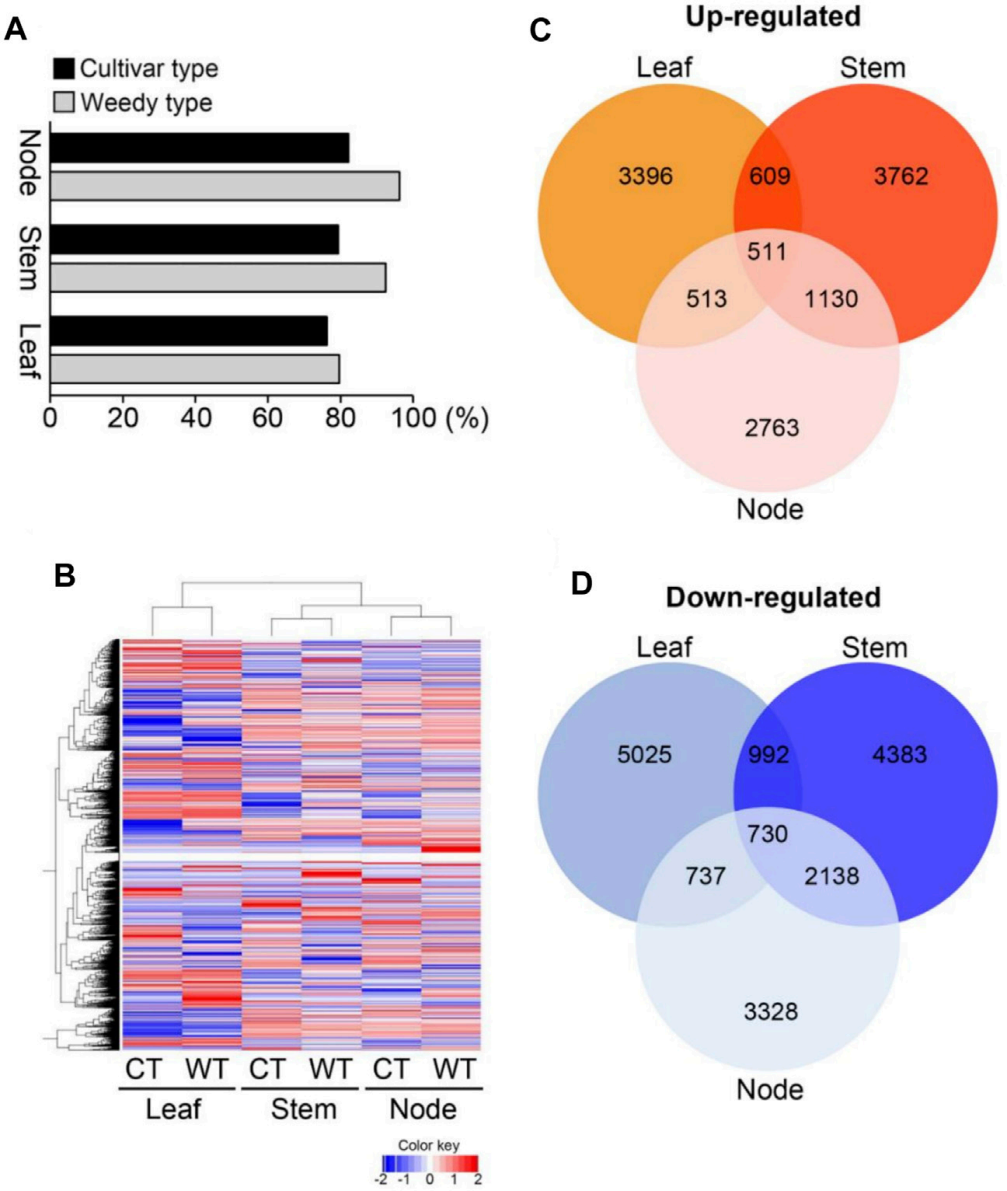
Transcriptome analysis of leaves, stems, and nodes of weedy type (WT) and cultivar type (CT) soybean. Total RNA was prepared from leaves, stems, and nodes of 12-week-old plants. **(A)** Mapping of sequence reads to the reference *Glycine max* Wm82.a2. v1. **(B**) Hierarchical cluster analysis of differentially expressed genes (DEGs). Color key indicates gene expression FPKM values from −2 to 2: blue = lowest and red = highest among the DEGs between leaves, stems, and nodes of WT and CT. **(C)** and **(D)** Comparisons of the number and overlapping relationship of the DEGs between leaves, stems, and nodes of WT and CT. Red circles indicate up-regulated genes **(C)** and blue circles represent down-regulated genes **(D)** in cultivar type with respect to weedy type (>log_2_ fold change).

To identify differences in development-related gene expression in RILs, we analyzed the transcriptomes of the leaves, stems, and nodes of weedy and cultivar growth type plants obtained in F_7_ using RNA sequencing (Figure 2; Table 1). There were 224,804,226 sequencing reads in the raw data and 213,047,794 reads in the clean data (94.77% of the total) (Table 1). The trimmed sequence reads were mapped to the reference genome (*Glycine max* Wm82. a2. v1), and the average percentage of mapped reads amongst all the samples was 84.69%. The average ratios of mapped reads in stems, nodes, and leaves of weedy and cultivar type plants were 85.5%, 89.17%, and 79.4%, respectively (Figure 2A). A total of 73,722 unigenes were generated for DEG analysis. The results indicate that the quality of sequencing was satisfactory for further analysis.

**TABLE 1:** The summary of the sequencing data by a high-throughput DNA sequencing platform in the weedy type and cultivar type soybeans.

Sample name	Raw Data		Trimmed Data		
	Total reads	Total length (bp)	Total reads	Total length (bp)	Mapped reads
Leaf (Weedy)	38,985,736	5,886,846,136	37,622,202	5,555,589,385	29,947,496
Leaf (Cultivar)	37,163,952	5,611,756,752	34,725,720	5,110,124,789	26,480,436
Stem (Weedy)	33,235,950	5,018,628,450	31,570,310	4,636,873,435	29,214,016
Stem (Cultivar)	34,983,866	5,282,563,766	33,076,282	4,863,194,941	26,226,106
Node (Weedy)	34,844,032	5,261,448,832	33,229,264	4,891,544,135	31,957,634
Node (Cultivar)	45,590,690	6,884,194,190	42,824,016	6,279,165,817	35,191,134

### DEG Analysis Between Weedy and Cultivar Type Plants

To investigate the gene expression levels of each sample, the transcripts were assembled and normalized by fragments per kilobase of transcript per million mapped reads (FPKM), and DEGs in each weedy type vs. cultivar type pair (leaves, stems, and nodes) were selected with *p*-value < 0.05 and | log_2_ (fold change) | ≥ 1. A total of 38,618 DEGs were visualized using a heat map (Figure 2B). The heat map showed that the color of the plots for stems and leaves was more visually different compared to the nodes of weedy type and cultivar type plants. A total of 12,513, 14,255, and 11,850 DEGs in leaves, stems, and nodes, respectively, were screened. Specifically, 5,029, 6,012, and 4,917 upregulated genes and 7,484, 8,243, and 6,933 downregulated genes were identified in leaves, stems, and nodes, respectively (Table 2). Furthermore, the Venn diagram of DEGs showed that 609, 513, and 1,130 upregulated genes (Figure 2C) and 992, 737, and 2,138 downregulated genes (Figure 2D) overlap in leaves and stems, leaves and nodes, and stems and nodes, respectively. Also, 511 upregulated genes and 730 downregulated genes overlapped in all samples (leaves, stems, and nodes) (Figures 2C,D). We constructed a Minus-versus-Add (MA) plot and a Volcano plot of the DEGs, which showed that the stem of the weedy and cultivar types had the greatest number of DEGs (Supplementary Figure S2), suggesting that the stem has more contribution for phenotype variation in plants. The analyses indicate that gene expression changes are involved in the characteristic developmental differences of weedy type and cultivar type plants.

**TABLE 2:** Status of differentially expressed genes between weedy and cultivar types (*p*-value < .05).

Samples	*p* val<.05, LFC>=2		
	Up regulated	Downregulated	Total
Leaf (Weedy vs. Cultivar)	5029	7484	12513
Stem (Weedy vs. Cultivar)	6012	8243	14255
Node (Weedy vs. Cultivar)	4917	6933	11850

**FIGURE 3:**
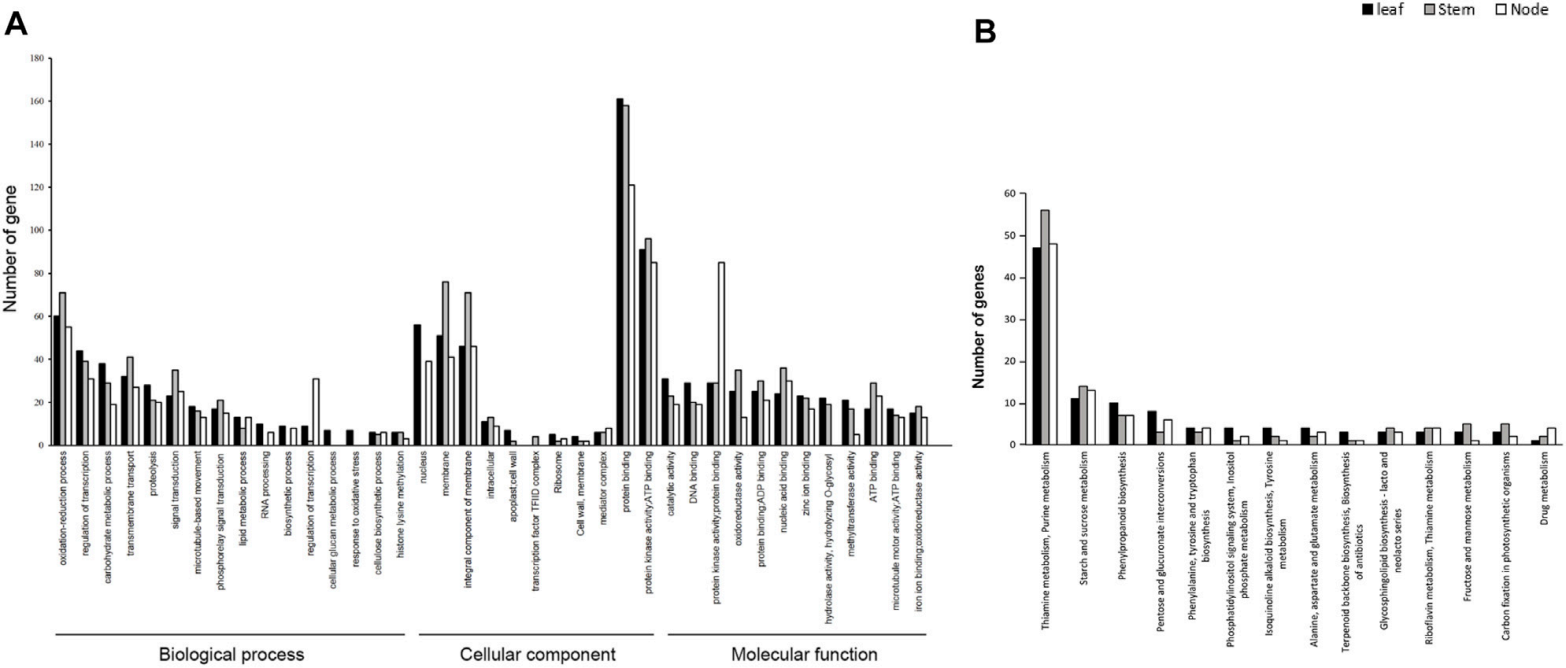
Gene Ontology (GO) functional classification and Kyoto Encyclopedia of Genes and Genomes (KEGG) classifications of DEGs of leaves, stems, and nodes. **(A)** GO analysis of differentially expressed genes (DEGs). GO describes number of most enriched DEGs with three independent categories: biological process, cellular component, and molecular function. The x-axis represents the GO terms and the y-axis the number of unigenes. **(B)** KEGG pathway of DEG terms grouped into 15 categories. The x-axis represents KEGG terms and the y-axis number of unigenes.

Pathway Enrichment Analysis of DEGs for GO Annotation and KEGG. To determine the associations of DEGs in the development of leaves, stems, and nodes in weedy and cultivar type soybeans, we performed GO annotation and KEGG pathway enrichment analysis using DEGs. A total of 73,722 DEGs were annotated in three major categories: biological processes, cellular components, and molecular function (Figure 3A). The total of 21,618, 8,107, and 33,821 were assigned to biological process, cellular component, and molecular function, respectively. The molecular function category showed a greater number of DEGs compared to the other categories. A total of 12,513 DEGs in leaves, 14,255 DEGs in stems, and 11,850 DEGs in nodes were classified into GO terms. Of the total, 1,574 DEGs in leaves, 4,762 DEGs in stems, and 3,866 DEGs in nodes were classified as being involved in biological processes; 554 DEGs in leaves, 1,619 DEGs in stems, and 1,407 DEGs in nodes were classified as being involved in cellular components; and 2,401 DEGs in leaves, 7,119 DEGs in stems, and 5,865 DEGs in nodes were classified as being involved in molecular function. Among them, the number of DEGs in stems was significantly enriched in GO terms. The enriched DEGs in stems are associated with protein phosphorylation, oxidation-reduction, transcription factor regulation, carbohydrate metabolism, and transmembrane transport in biological process; with nucleus, membrane, and integral components of membranes in cellular component; and with catalytic activity, DNA binding, protein binding, and protein and nucleic acid binding in molecular function. These GO terms might be related to the phenotype of weedy type and cultivar type plants.

To better understand the molecular difference between weedy and cultivar types, the upregulated and downregulated DEGs were grouped by KEGG. The pathways of the DEGs were analyzed using the KEGG database (http://www.genome.jp/kegg/pathway.html) (Figure 3B). A total of 814 unigenes were annotated to various pathways, of which 159, 172, and 149 DEGs of leaves, stems, and nodes, respectively, were assigned to a KEGG pathway. The KEGG classification showed that the DEGs were mainly involved in thiamine metabolism in leaves, stems, and nodes. For DEGs in the leaf, 47 were assigned to thiamine and purine metabolism, 11 to starch and sucrose metabolism, and 10 to phenylpropanoid biosynthesis. In DEGs in the stem, 56 were assigned to thiamine and purine metabolism, 14 to starch and sucrose metabolism, and 7 to phenylpropanoid biosynthesis. For DEGs in the node, 48 were assigned to thiamine and purine metabolism, 13 to starch and sucrose metabolism, and 7 to phenylpropanoid biosynthesis. Unlike in the leaves and nodes, 14 DEGs of the stems were highly enriched in starch and sucrose metabolism and porphyrin and chlorophyll metabolism. These pathways were related to differences in weedy type and cultivar type plant height, indicating that several genes regulate the growth of soybean, through controlling biological processes.

### Identification of Genes Related to Traits Between Weedy and Cultivar Type Soybeans

The above results suggest that several genes play important roles in the antagonistic regulation of plant development to produce weedy type and cultivar type plants. The development of leaves, stems, and nodes is regulated by the plant hormones gibberellin, auxin, and cytokinin. We analyzed and compared the DEGs that respond to gibberellin (GA), auxin, and cytokinin in the leaves, stems, and nodes (Table 3). The GA-related genes were upregulated in the leaves, stems, and nodes of the cultivar type plant compared to the weedy type of plant. *SPINDLY* (*SPY*) and Gibberellin 2-oxidase (*GA2Ox8*) genes were upregulated in the cultivar type plant. Homeobox protein (*HAZ1*) and Kaurene Oxidase (*KO*) genes were down regulated in the leaves, stems, and nodes of the cultivar type compared to the weedy type. These genes are related to the gibberellin signaling pathway that regulates the level of GA. The overexpression of *SPY* and *GA2Ox8* in *A. thaliana* showed reduced plant height and thick stems when compared to control (Thornton et al., 1999). These results indicate that upregulation of *GA2ox* in plants leads to short and thick stem. Most of the auxin-related genes are downregulated in cultivar type soybean. Auxin response factor 5 (*ARF5*) and FERONIA (*FER*) showed remarkable upregulation, whereas *POPCORN* (*PCN*), PIN like proteins (*PINS*), Auxin response element (*AUX22E*), Indole Acetic acid (*IAA30*), and Auxin response factor (*ARF8*) were downregulated in leaves, stems, and nodes of the cultivar type compared to the weedy type. These genes are known as auxin signaling genes. Reduction in *FER* expression in *A. thaliana* results in reduced cell elongation in various tissues (Guo et al., 2009). These genes play a prominent role in producing a thick stem in the cultivar type and a thin stem in the weedy type in soybean. Cytokinin dehydrogenase-1 (*CKX1*), Arabidopsis Histidine kinase (*AHK3*), Arabidopsis histidine phosphotransfer (*AHP1* and *AHP5*), Arabidopsis response regulator (*ARR2*), and *KORRIGAN* (*KOR*) are up regulated in the cultivar type compared to the weedy type. Overexpression of cytokinin response gene (*CKX*) in *A. thaliana* results in shorter internodes with reduced growth (Werner et al., 2001), and changes in the expression of cytokinin response genes result in variation of meristem size (Schmülling et al., 2003). The expression of the regulator and receptor genes in the stem of weedy and cultivar type, response to the major phytohormones is shown in Supplementary Table S1. The catalyzing enzyme of Gibberellin, cytochrome P450, called *ent*-kaurenoic acid oxidase (KAO) were up regulated in the stem of weedy type. Similarly, the DELLA proteins response to GA signaling were up regulated in the stem of cultivar types. DELLA proteins are the negative regulator of gibberellin signaling (Yoshida et al., 2014). Most of the auxin and cytokinin response regulatory genes are up regulated in the stem of cultivar types (Supplementary Table S1). These findings show that the phytohormone response genes play an important role in the stem of the shoot compared to the leaf and node.

**TABLE 3:** Significantly differentially expressed gene response to phytohormones in cultivar types with respect to weedy types.

Phytohormone	Gene name	Ascession No	Fold change		
			Leaf	Stem	Node
Gibberellin	Spindly (*SPY*)	Glyma.02G201300.4	7.32	9.83	10.32
	Gibberellin 2-oxidase (*GA2Ox8*)	Glyma.17G178300.2	6.83	7.17	7.54
	*GA2Ox8.2*	Glyma.13G287600.1	11.43	2.53	4.21
	Homeobox protein (*HAZ1*)	Glyma.17G210100.1	−4.03	−3.22	−5.85
	Kaurene oxidase (*KO*)	Glyma.14G219100.1	−3.54	−1.81	−2.16
Auxin	Auxin regulator factor (*ARF5*)	Glyma.01G002100.2	4.16	5.72	4.76
	FERONIA (*FER*)	Glyma.19G033100.1	8.23	8.36	5.65
	PIN-LIKES (*PILS3*)	Glyma.16G069400.2	−4.34	−9.84	−11.11
	Peroxisomal ABC transporters (*ABCD1*)	Glyma.09G043000.1	−11.06	−7.45	−1.66
	LIKE AUXIN protein (*LAX1*)	Glyma.17G065000.1	−5.04	−4.09	−2.40
	POPCORN (*PCN*)	Glyma.16G017600.2	1.69	−3.99	−9.01
	FERONIA (*FER*)	Glyma.11G141500.2	−1.84	−3.23	−1.75
	Auxin response element (*AUX22E*)	Glyma.14G156300.1	1.80	−1.73	−1.32
	Indole acetic acid (*IAA30*)	Glyma.17G082700.1	1.58	−1.64	−1.39
	Auxin response factor (*ARF8*)	Glyma.20G139100.1	−3.40	−1.61	−5.37
Cytokinin	Arabidopsis histidine kinase (*AHK3*)	Glyma.08G105000.5	7.14	7.14	3.54
	Cytokinin dehydrogenase (*CKX1*)	Glyma.03G133300.1	2.85	6.82	2.58
	Arabidopsis histidine phosphotransfer (*AHP1*)	Glyma.10G157800.1	6.55	3.49	2.29
	*AHP5*	Glyma.02G150800.2	2.23	3.47	2.35
	Arabidopsis response regulator (*ARR2*)	Glyma.07G079000.1	5.20	2.75	4.71
	KORRIGAN (*KOR*)	Glyma.06G123900.1	2.30	2.12	3.43

### Validation by Reverse-Transcription Quantitative PCR

**FIGURE 4:**
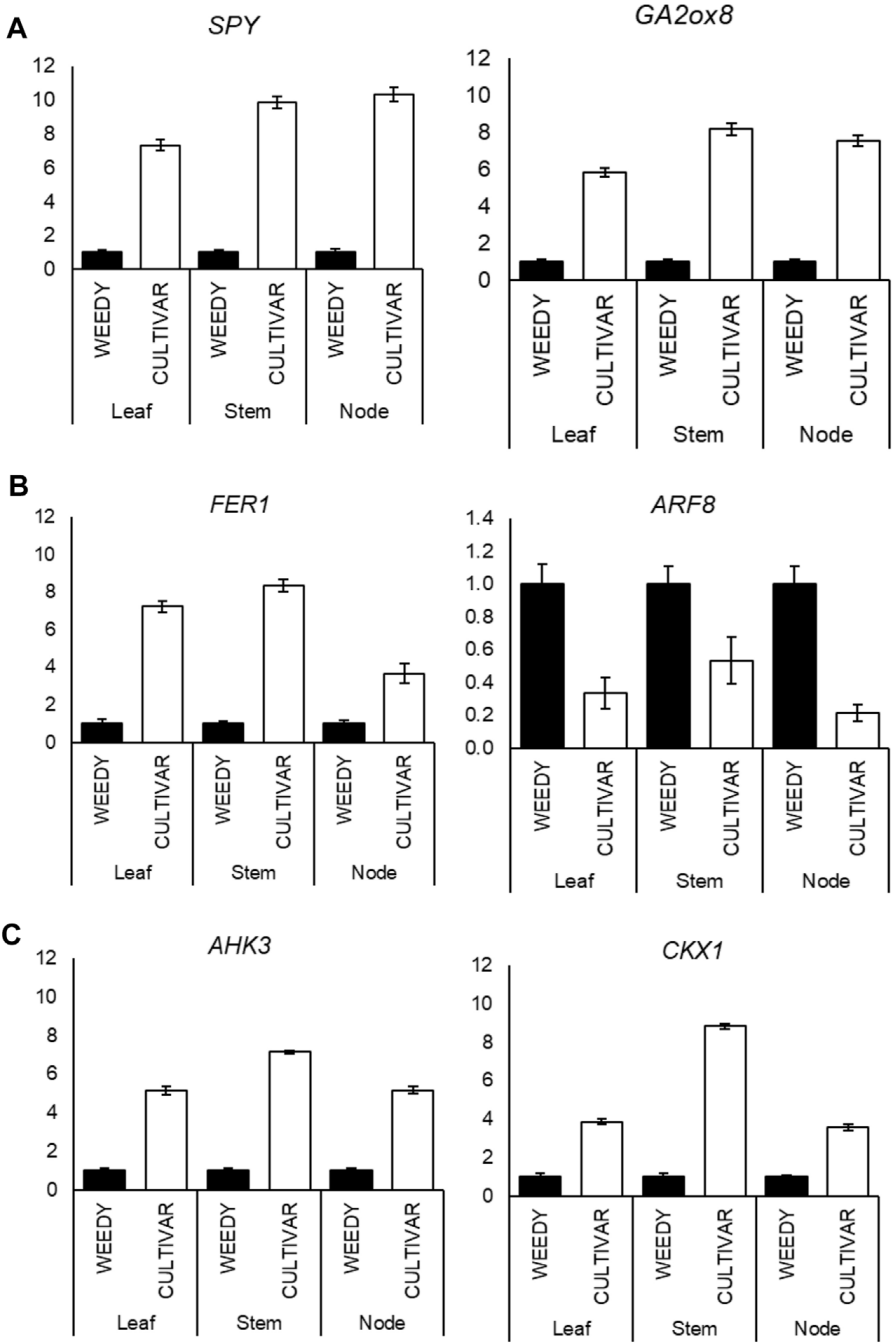
**(A–C)** Reverse-transcription quantitative PCR (RT-qPCR) analysis shows changes in gibberellin response genes: gibberellin 2-oxidase 8 (*GA2ox8*) and SPINDLY (*SPY*) **(A)**; auxin response genes: FERONIA (*FER*) and auxin response factor (*ARF8*) **(B)**; and cytokinin response genes: Arabidopsis histidine kinase-3 (*AHK3*) and cytokinin dehydrogenase-1 (*CKX1*). **(C)** Expression levels in the leaves, stems, and nodes of weedy type (WT) and cultivar type (CT). Total RNA was prepared from the 12-week-old plants for the indicated samples. Control plants were weedy type (WT). Error bars indicate SD of triplicate measurements. *GmAct6* was used as internal control, and relative expression levels are shown as fold values.

To test the reliability of FPKM expression patterns (RNA-Seq) of the selected DEGs, we checked the expression level by RT-qPCR. A total of 22 DEGs that respond to plant phytohormones were selected from the weedy and cultivar types. The genes were classified under the three major plant hormones: gibberellin, auxin, and cytokinin (Figure 4; Table 3). Most of the gibberellin response genes were found to be up regulated in the cultivar type with respect to the weedy type. The expression of GA response genes, *SPY* and *GA2ox*, was found to be significantly higher in the stem of the cultivar type compared to the weedy type (Figure 4A). The expression of genes encoding Homeobox protein (*HAZ*1) and Kaurene oxidase (*KO*) was down regulated in the cultivar growth type in soybean (Supplementary Figure S1). Based on the RT-qPCR, auxin response genes were downregulated in the leaves, stems, and nodes of the cultivar type relative to the weedy type. Here, the auxin response gene *FER1* was upregulated and *ARF5* was downregulated in the cultivar type compared to the weedy type (Figure 4B). Cytokinin-responsive genes were found to be upregulated in cultivar types. *AHK3* and *CKX1* showed higher expression in the leaves, stems, and nodes of the cultivar type with respect to the weedy type (Figure 4C). According to the RT-qPCR expression and transcriptome data, we found that auxin and cytokinin are antagonistically expressed in leaves, stems, and nodes among cultivar and weedy type soybeans. The expression profile of the remaining genes is described in Supplementary Figures S1A–C. Here, we focused on the stem phenotype, and the genes studied were most differentially expressed in the stem compared to the leaf and the node of weedy and cultivar type soybean. The genes and their expression patterns were mostly consistent with transcriptome data; hence, the results indicate the transcriptome data we presented were reliable and accurate.

## Discussion

Plant morphology and architecture are an important economic characteristic that impact soybean yield. Although several factors that affect soybean growth and development are known, many aspects of this process remain to be elucidated. Therefore, studying gene expression and function with respect to phenotype provides an efficient way to reveal the hidden mechanism. In addition, comparative RNA sequencing technology allows us to investigate the molecular mechanism between two plant relatives. In this study, we performed a transcriptome analysis between weedy and cultivar growth types in soybean obtained from the F_7_ RIL population derived from a cross of wild and cultivated soybean. Our findings show that the weedy type has a longer stem and creeps on the ground, whereas the cultivar type exhibits the upright standing growth type. From the result, we hypothesize the characteristic of the stem determines if the plant is the weedy or cultivar type in soybean. Furthermore, the stem transcriptome was investigated to understand the relationship between gene regulation and phenotype.

Phytohormones participate in many physiological processes in plants (Bari and Jones, 2009; Luo et al., 2016). Auxin, gibberellin, cytokinin, and ethylene play abundant roles in shaping the morphological structure of plants (Fahad et al., 2015). Gibberellin 2-oxidase (*GA2ox*) is involved in degradation of bioactive GA and controls GA levels (Martin et al., 1999; Thomas et al., 1999). *GA2ox* modulate under the GA biosynthesis and inactivation pathway which converts the active form of GAs to inactive form (Yamaguchi, 2008). Upstream of *GA2ox* inactivates bioactive GA resulted in short internode plants whereas its downstream induce stem elongation (Supplementary Figure S3). Overexpression of two switchgrass *GA2ox* genes showed abnormal shoot architecture with extremely reduced internodes (Wuddineh et al., 2015). Similarly, overexpression of *A. thaliana GA2ox8* causes a decrease in GA levels, which showed a reduction in stem length (Schomburg et al., 2003). Additionally, *GA2ox1* transformed *Solanum* species exhibited low GA with phenotype alteration, reduced plant height, and internode distance (Dijkstra et al., 2008). Downstream of GA level in plants results in semi dwarf, while increased GA concentration promote taller plants (Busov et al., 2008). Elongated stem and vine growth habit are characteristic of the wild phenotype in soybean (Tian et al., 2010). In our study, the cultivar type has short internodes and thick stems, whereas the weedy type has thin and long stems (Figure 1C). RNA-Seq data revealed that *GA2ox8* is differentially expressed between weedy and cultivar types of soybeans. The expression of *GA2ox8* was found to be up regulated in the stem of the cultivar type, which might reduce the GA level and result in a short and thick stem. Conversely, the expression of *GA2ox* was down regulated in weedy type, which resulted in a thin stem with long internodes. The RT-qPCR analysis showed that the transcription level of *GA2ox* in the cultivar type was higher in comparison to the weedy type (Figure 4A). SPINDLY (SPY) is the GA-signaling protein that also functions as a negative regulator of GA during seed germination (Qin et al., 2011) and is characterized by slender phenotype (Martin et al., 1999) Altered *SPY* expression affects overall plant growth (Swain et al., 2001). Thus, the mutant plant lacking *SPY* showed suppressed phenotype plants (Thornton et al., 1999). Here, *SPY* is upregulated in the cultivar type plant and downregulated in the weedy type of plant (Figure 4A). This indicates that deregulation of *SPY* results in an altered phenotype. Additionally, *SPY* genes positively interact with cytokinin, which plays a major role in plant growth and development (Greenboim-Wainberg et al., 2005). The activity of cytosolic SPY stimulates cytokinin and inhibits GA signaling (Greenboim-Wainberg et al., 2005; Maymon et al., 2009). Two other GA response genes, homeobox protein (*HAZ1*) and kaurene oxidase (*KO*), are down regulated in cultivar type plants (Supplementary Figure S1A). *KO* also called cytochrome P450, catalyzes successive oxidation of enzymes in gibberellin biosynthesis pathway (Morrone et al., 2010), (Supplementary Figure S3). Therefore, GA-responsive genes might contribute to phenotype variation to produce either the weedy or cultivar type.

Auxin regulates divergent developmental processes and helps in anisotropic cell expansion in plants (Kutschera et al., 1987; Zhao, 2010; Zazimalová et al., 2014). *ARF8* helps in cell expansion and proliferation during plant development (Varaud et al., 2011). *ARF8* is a positive regulator of auxin, and its overexpression resulted in a long hypocotyl in *A. thaliana* (Tian et al., 2004). Likewise, high expression of *ARF8* in tobacco enhanced plant growth and development (Ge et al., 2016). Sometimes, deregulation of *ARF8* causes developmental abnormalities in *A. thaliana* (Jay et al., 2011). *ARF8* showed a conserved role in controlling the vegetative growth and shoot development of tomato (Liu et al., 2014). Functional deficient mutant of *ARF8* in *Arabidopsis* showed auxin deficient phenotypes such as dwarfism than the wild types (Nagpal et al., 2005). Here, *ARF8* was upregulated in the weedy type of soybean and downregulated in the cultivar type soybean. This might enhance stem elongation in weedy type and reduce stem length in cultivar type. Furthermore, *ARF8* also promotes early flowering in *A. thaliana* (Nagpal et al., 2005; Goetz et al., 2007; Finet et al., 2010). In agreement with previous studies, the weedy type plants were observed to mature earlier compared to the cultivar type plants (Figure 1A). *FERONIA* (*FER*) is one of the auxin response genes known as a cell growth regulator (Shih et al., 2014; Oh et al., 2020). A study of an *A. thaliana FER* mutant showed that FER is essential for maximum cell elongation and proliferation (Guo et al., 2009). In addition, *FER* interacts with components of the cell wall to assist in monitoring of the cell walls in plants (Shih et al., 2014). In this study, *FER* was up regulated in the cultivar type compared to weedy type, indicating that more expansion of cells might result in thick stems in cultivar plants. RT-qPCR results showed that *FER* is highly expressed in cultivar plants (Figure 4B). There was differential expression in other auxin response genes as well; auxin response factor 5 (*ARF5*) was upregulated in the cultivar type, whereas *PIN LIKES3* (*PILS3*) was downregulated in the cultivar type (Supplementary Figure S1B). PILS are the putative auxin carriers, its overexpression affects overall plant patterning (Feraru et al., 2012), while mutant leads to reduced plant growth in *Arabidopsis* (Barbez et al., 2012). A study has shown that *ARF5* interacts with *PIN1* in the formation of leaf primordia (Schuetz et al., 2019).

The Cytokinin oxidase/dehydrogenase (CKX) gene family regulates cytokinin in plants (Wang et al., 2014), playing a major role in metabolic cytokinin inactivation (Motyka et al., 1996; Mok and Mok, 2001). Overexpression of *A. thaliana CKX1* resulted in short internodes with reduced growth compared to control (Werner et al., 2001). Also, similar results were obtained in transgenic tobacco, where a higher expression of *CKX1* decreased the cytokinin level and resulted in reduced plant height (Yang et al., 2003). Here, *CKX1* was found to be up regulated in cultivar type plants compared to weedy type plants, indicating that *CKX1* plays a major role in making cultivar type plants exhibit thick stems and shorter plant height compared to the weedy type. Cytokinin signaling pathway consists of three proteins, histidine kinase receptor, histidine phosphotransfer and response regulator (Keshishian and Rashotte, 2015). Arabidopsis histidine kinase (*AHK3*) is an important cytokinin receptor responsible for secondary growth of vascular tissues (Hejátko et al., 2009). Loss of *AHK3* results in reduction of overall shoot growth (Higuchi et al., 2004). This suggests that *AHK3* is essential for overall shoot formation. Our transcriptome and expression analysis showed that *CKX1* and *AHK3* are highly expressed in the shoots of cultivar type plants (Figure 4C). Overexpression of Arabidopsis response regulator (*ARR2*) was shown in a previous study to result in dwarf plants and induce a cytokinin hypersensitive response (Shull et al., 2016). (Hwang and Sheen, 2001) reported ARR type-A proteins act as negative regulator of cytokinin signaling. Here, *ARR2* was up regulated in the cultivar type (Supplementary Figure S1C). This result suggests that cytokinin signaling genes might be responsible for varying overall shoot morphology in plants.

Interaction of major plant hormones influence growth and development, which can elucidate the genetic basis and molecular mechanisms behind changes in plant physiology (Ross and O’Neill, 2001). Hormone interactions can increase or decrease the expression of responsive genes (Vanstraelen and Benkov, 2012). Auxin and gibberellins are essential plant hormones that perform different functions but exist at an equilibrium for normal stem elongation (Yang et al., 1996; Haga and Iino, 1998). In Chinese cabbage, an increase in biosynthesis of GA and indole acetic acid (*IAA*), which is an auxin, promotes stalk elongation (Kou et al., 2021). GA coordinates with auxin for cell division and growth of cambial derivatives in Poplar stems (Björklund et al., 2007). In gibberellin biosynthesis pathway, the biosynthesis of GA activates with the regulatory genes and interacts coordinately with the other phytohormone response genes which results in morphology variation (Supplementary Figure S3). We predict that the interaction of auxin and GA response genes in weedy type plants results in longer and thin stems compared to the cultivar type. In various developmental processes, gibberellin and cytokinin have opposite effects but are coordinately expressed (Greenboim-Wainberg et al., 2005). The *SPY* gene inhibits bioactive GA but promotes the cytokinin signaling pathway (Filardo and Swain, 2003; Eckardt, 2005). Cytokinin regulates auxin signaling metabolism; thus, higher expression of cytokinin response genes promotes the expression of auxin response genes (El-Showk et al., 2013). The expression of auxin is indirectly inhibited by the cytokinin signaling mechanism when cytokinin biosynthesis increases (Jasinski et al., 2005; Yanai et al., 2005). Arabidopsis response regulators (ARRs) work together to influence cytokinin and auxin signaling, which are the primary regulatory mechanisms at the shoot apical meristem (Zhao et al., 2010). DELLA proteins participate in complex crosstalk among plant hormone through interaction with many transcription factors (Hou et al., 2010; Yoshida et al., 2014; Li et al., 2015). The outcome of auxin and cytokinin interaction depends upon different cell-type specifications via different signaling pathways (Chandler and Werr, 2015). By means of synergistic or antagonistic action, phytohormones interact with developmental cues along with delivering environmental inputs as signaling crosstalk (Seif El-Yazal et al., 2015).

## Conclusion

In summary, our study postulates candidate genes responsible for the weedy and cultivar growth types in soybean. Using the Illumina platform, transcriptome sequencing was performed and DEG analysis revealed that major phytohormone-responsive genes influence plant growth types. By comparing the gene expression of the shoots (leaves, stems, and nodes) of weedy and cultivar types, we found that the stem is the key modulator of phenotype variation in the F_7_ RIL population. *GA2ox*, *SPY*, *ARF*, *CKX*, and *AHK* are the major plant hormone signaling genes responsible for determining the weedy and cultivar growth types in soybean. These results show how the expression, regulation, and interaction of major plant hormone-responsive genes influences plant phenotype. Our study provides insights for future genetic breeding and facilitates target trait crop improvement for higher yield in soybean.

## Data Availability

The datasets presented in this study can be found in online repositories. The names of the repository/repositories and accession number(s) can be found below: https://www.ncbi.nlm.nih.gov/geo/query/acc.cgi?acc=GSE188519.

## Supplementary Material

Supplementary Figure 1 Reverse-transcription quantitative PCR (RT-qPCR) analysis shows changes in gibberellin response genes: gibberellin 2-oxidase 8 (*GA2ox8.2*), Homeobox protein (*HAZ1*), and Kaurene oxidase (*KO*) **(A)**; auxin response genes: Auxin response factor 5 (*ARF5*), *PIN LIKES3* (*PILS3*), LIKE AUXIN protein (*LAX1*), Indole acetic acid 30 (*IAA30*), and Auxin response element (*AUX22E*) **(B)**; and cytokinin response genes: Arabidopsis histidine phosphotransfer 1 (*AHP1*), Arabidopsis histidine phosphotransfer 5 (*AHP5*), Arabidopsis response regulator (*ARR2*), *KORRIGAN* (*KOR*), and Arabidopsis Histidine Kinase (*AHK3*) **(C)**. Expression levels in leaves, stems, and nodes of weedy and cultivar types. Total RNA was prepared from the 12-week-old plants for the indicated samples. Control plants were weedy type (WT). Error bars indicate SD of triplicate measurements. *GmAct6* was used as internal control, and relative expression levels are shown as fold values.

Supplementary Figure 2 Expression profiles of the DEGs using Volcano and Minus-versus-Add (MA) plots. **(A)** Volcano plot of the upregulated and downregulated DEGs of the stems between the weedy and cultivar growth types. The down-regulated DEGs are on the left side, and up-regulated DEGs are on the right. Red indicates DEGs with *p*-value < .01 and LFC ≥ 1; green indicates *p*-value between .01 and .05, and LFC ≥ 1; yellow indicates *p*-value < .01 and LFC ≥ 1; and black indicates *p*-value < .05 and LFC ≤ 1. For each plot, the x-axis represents the log_2_ fold change (LFC), and y-axis represents −log 10 (*p*-values). **(B)** MA plot of the DEGs of the stem between the weedy and cultivar growth types. The x-axis represents the mean of the normalized counts of the genes, and the y-axis indicates the log_2_ fold change. Red points represent up-regulated DEGs, blue points represent down-regulated DEGs, and black points represent non-DEGs.

Supplementary Figure 3 Relevant part of GA biosynthesis pathway and crosstalk. Solid boxes indicate the bioactive form of the GA, and the names with arrow headed are the catalyzing enzymes. The dashed box and line represent the inactive form of the GA. The upward headed arrow represents up regulation of the phytohormone response genes. After the subsequent process and interaction with other phytohormone response genes results into bioactive and inactivate GAs. (*GA2ox*- Gibberellin 2 oxidase, *GA3ox* – Gibberellin 3 oxidase, *ARF* – Auxin response factor, *CKX* – Cytokinin dehydrogenase, PILS – PIN Like proteins, KAO – ent-kaurenoic acid oxidase). Interaction of these genes in GA pathway results in morphology variation in soybean plants. Adapted and modified from (Ross et al., 2001; Yamaguchi, 2008; Nomura et al., 2013).
