# Supplementary figures and images for: Identification and Characterization of Key Genes Responsible for Weedy and Cultivar Growth Types in Soybean
Source: Front Genet. 2022 Feb 24;13:805347. doi: 10.3389/fgene.2022.805347 (PMC8907156; doi:10.3389/fgene.2022.805347)

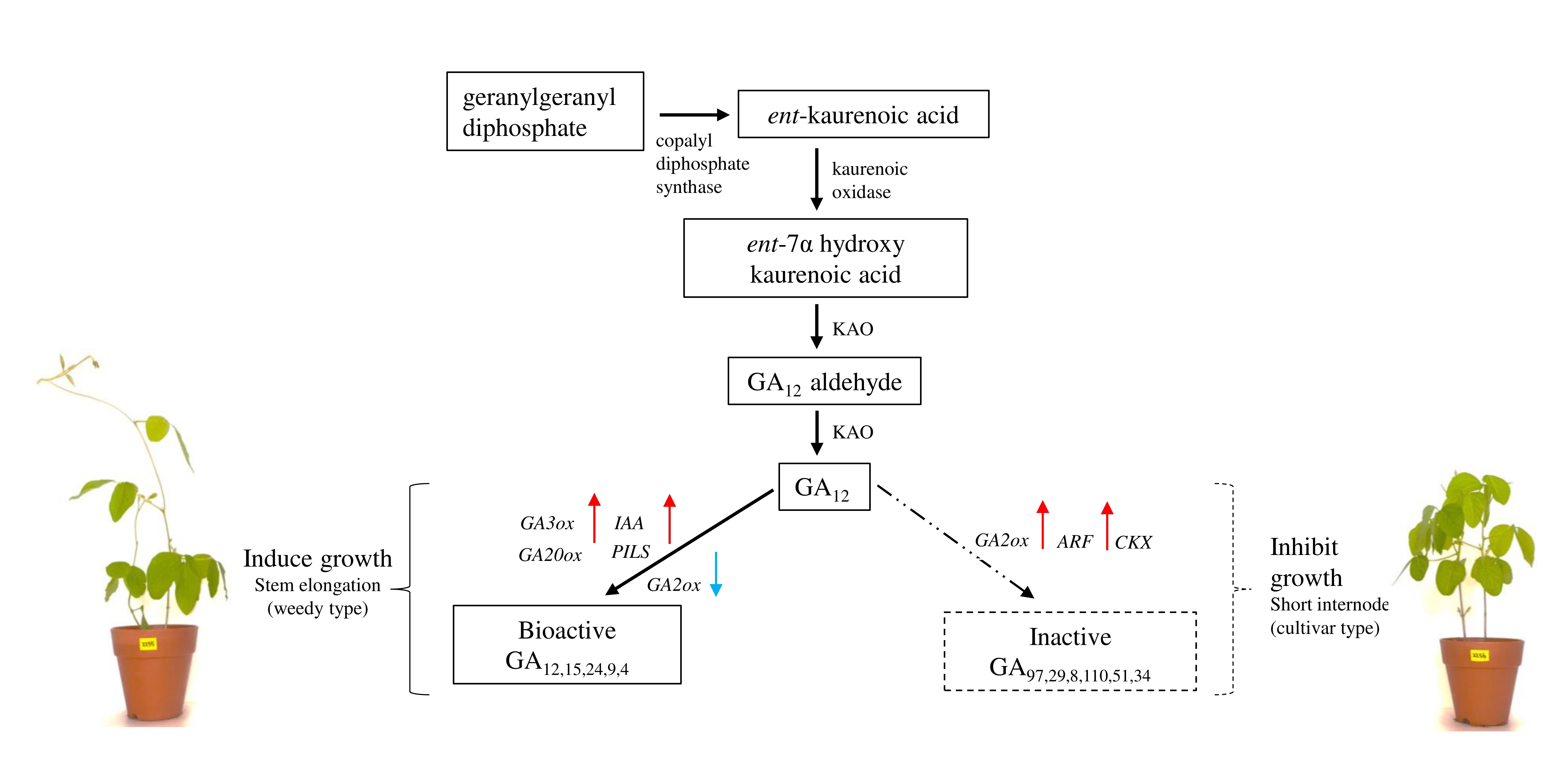

Supplement: Supplementary file 1 [file Image3.JPEG]

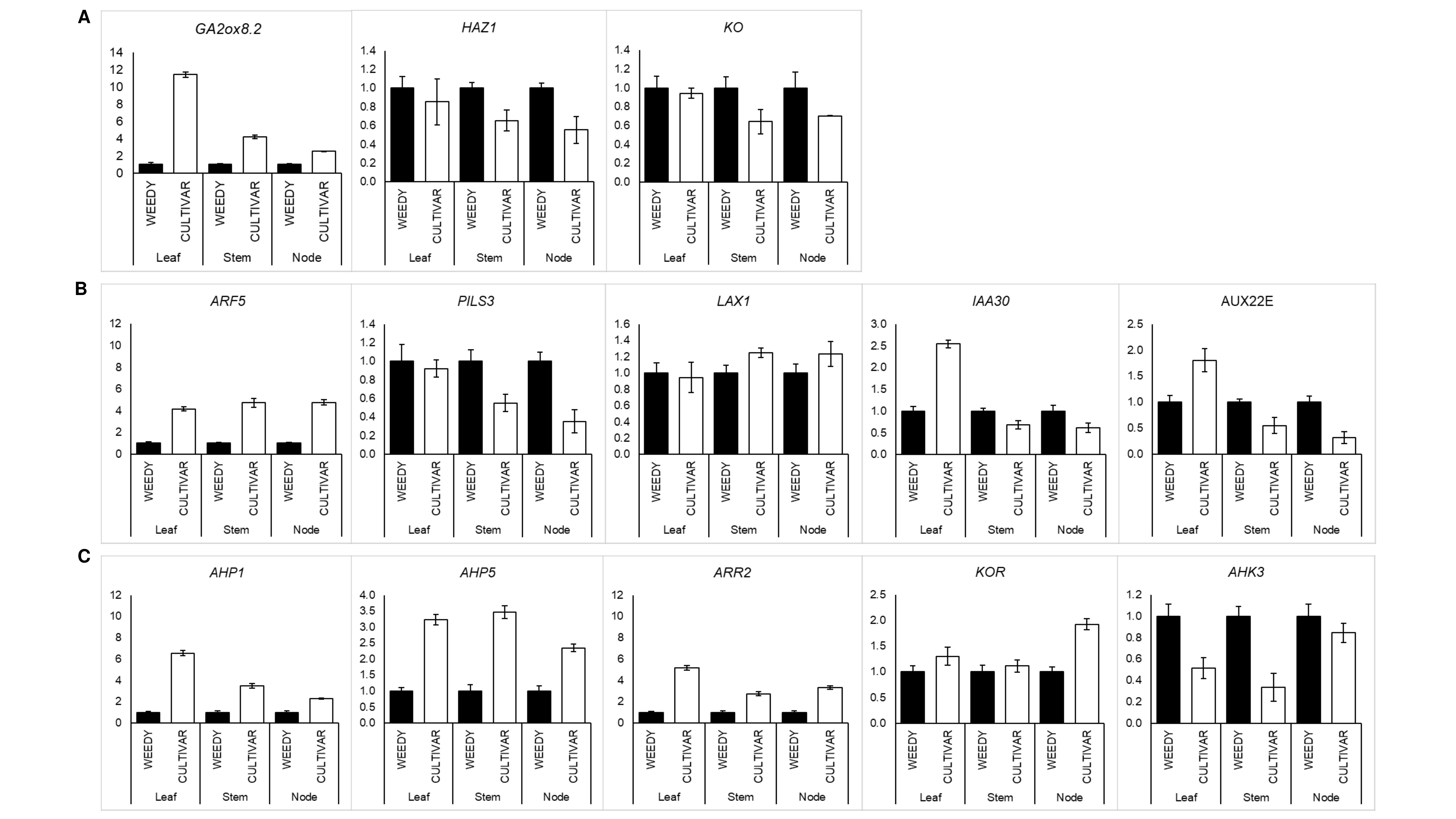

Supplement: Supplementary file 2 [file Image1.JPEG]

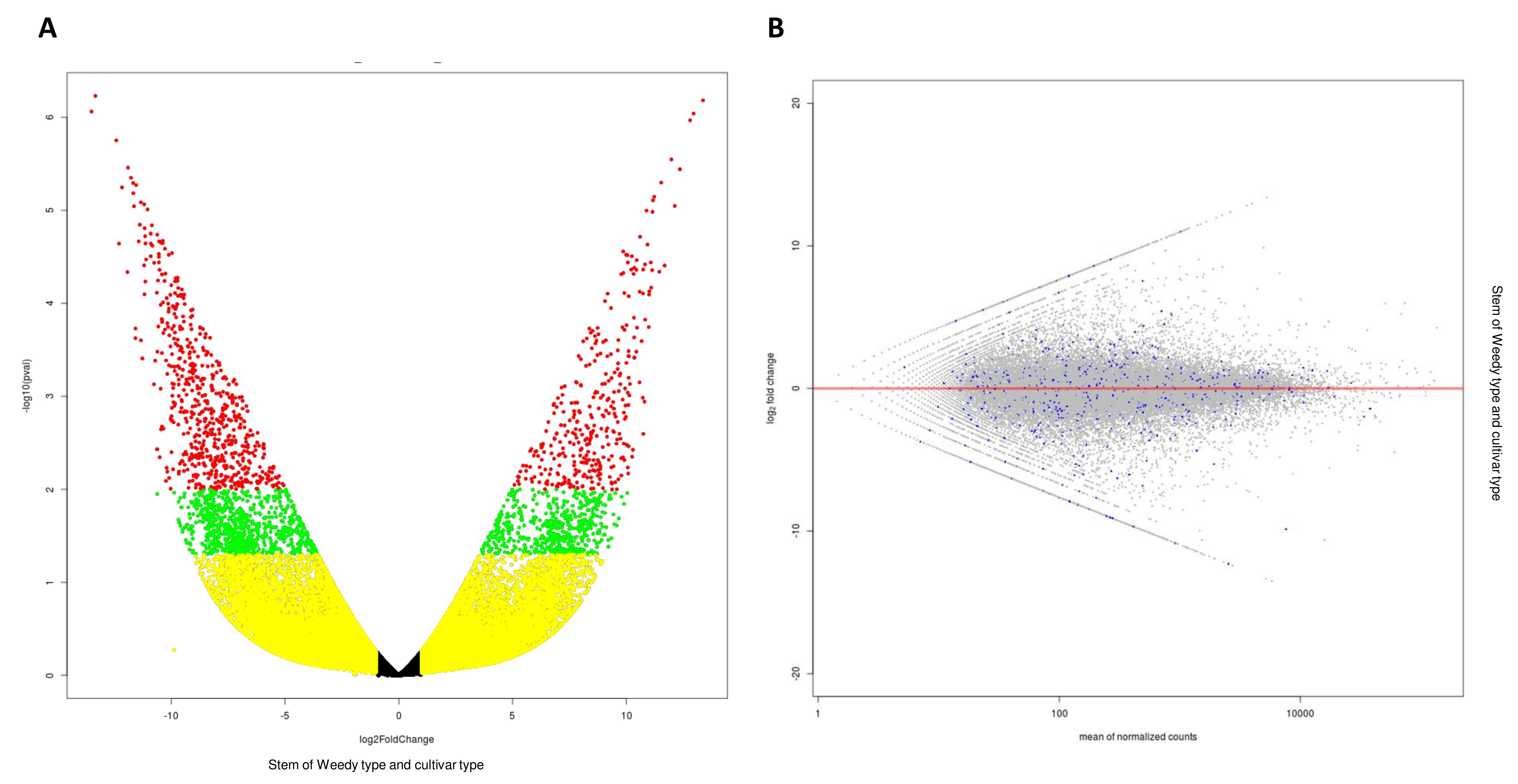

Supplement: Supplementary file 3 [file Image2.JPEG]
